# Genotype-associated heritable rumen bacteria can be a stable microbiota passed to the offspring

**DOI:** 10.1093/ismeco/ycad020

**Published:** 2024-01-10

**Authors:** Xinwei Zang, Huizeng Sun, Mingyuan Xue, Shulin Liang, Le Luo Guan, Jianxin Liu

**Affiliations:** Institute of Diary Science, College of Animal Sciences, Zhejiang University, Hangzhou 310058, China; Institute of Diary Science, College of Animal Sciences, Zhejiang University, Hangzhou 310058, China; Institute of Diary Science, College of Animal Sciences, Zhejiang University, Hangzhou 310058, China; Institute of Diary Science, College of Animal Sciences, Zhejiang University, Hangzhou 310058, China; Department of Agricultural, Food and Nutritional Science, University of Alberta, Edmonton, AB T6G 2P5, Canada; Faculty of Land and Food Systems, The University of British Columbia, Vancouver, British Columbia V6T 1Z4, Canada; Present address: Functional Genomics and Animal Microbiome, Faculty of Land and Food Systems, The University of British Columbia, MacMillan Bldg, 2357 Main Mall, Vancouver, BC V6T1Z4, Canada; Institute of Diary Science, College of Animal Sciences, Zhejiang University, Hangzhou 310058, China

**Keywords:** heritable rumen bacteria, compositional similarity, functions stability, dairy cows

## Abstract

Recent studies have reported that some rumen microbes are “heritable” (those have significant narrow sense heritability) and can significantly contribute to host phenotype variations. However, it is unknown if these heritable rumen bacteria can be passed to the next generation. In this study, the rumen bacteria from mother cows (sampled in 2016) and their offspring (sampled in 2019) were assessed to determine if vertical transmission occurred between the two generations. The analysis of relationship between host genotypes and heritable bacterial abundances showed that potential of five host genotypes can affect the relative abundances of two unclassified species level heritable bacteria (*Pseudoscardovia* and *p-251-o5*). The G allele of BTB-01532239 and A allele of ARS-BFGL-NGS-8960 were associated with a higher relative abundance of *p-251-o5*. The A allele of BTB-00740910 and BovineHD1300021786 and G allele of BovineHD1900005868 were associated with a higher relative abundance of *Pseudoscardovia.* The mother–offspring comparison revealed that the heritable rumen bacteria had higher compositional similarity than nonheritable bacteria between two generations, and the predicted heritable microbial functions had higher stability than those from nonheritable bacteria. These findings suggest that a high stability exists in heritable rumen bacteria, which could be passed to the next generation in dairy cows.

## Introduction

Linkage between rumen microbes and production traits of dairy and beef cattle, such as milk production and quality [(1, 2)] and feed efficiency [3, 4], has been widely reported recently. A recent study by Wallace et al. [5] identified 39 genera of core rumen bacteria that have significant heritability and showed significantly higher explanatory power for dairy cow’s phenotypes compared to other nonheritable core microbes. Similarly, our previous study found 32 heritable bacteria at species level and identified 63 host single nucleotide polymorphisms (SNP) associated with heritable bacteria [6]. In beef cattle, 59 rumen microbial taxa (at various classification levels) were identified to be heritable and 5 SNP were identified that were associated with both rumen heritable microbial taxa and feed efficiency traits [4]. Increasing evidence has shown that heritable bacteria were keystone members of rumen microbial interaction networks and are associated with various traits in beef [4] and dairy cattle [5], highlighting their roles in affecting microbial interactions and rumen functionality that related to production traits. Hence, manipulation of rumen heritable bacteria can be one of the effective strategies to optimize productive performance in cattle. Previous studies have primarily focused on the overall rumen microbiota, while the potential for greater phenotypic gains from manipulating heritable bacteria has not been assessed.

In cattle, the rumen microbiota undergoes dynamic changes throughout the growth process, and the development of rumen microbiome can have a direct effect on adult rumen microbiome [7]. In recent years, many studies revealed that calf’s gut microbiota can be traced back to their mother cows [8, 9]. For example, Klein-Jöbstl et al. [8] found that the mother cow’s vaginal microbiota was similar to those in the calf’s feces. Hence, the selection of adult cows and their offspring possessing mature and stable rumen microbiota would be more significant for investigating the composition and functionality of rumen microbiota in dairy cows. This is because the rumen microbiota in adult cows and their offspring exhibit a more stable composition and function compared to newborn or young cows. Our previous study revealed that heritable bacteria had a more significant average contribution to the lactation phenotype compared to nonheritable bacteria [6]. However, it remained unknown whether heritable bacteria can be transmitted from mother to offspring. We hypothesized that the host genotype could be associated with the rumen heritable bacterial relative abundance, and heritable rumen bacteria can be passed from cows to the offspring and have similar functions. Therefore, the objectives of this study were to identify the host loci that can affect heritable bacterial abundance and to investigate the composition and function of the rumen bacterial similarity between mother and their offspring.

## Materials and methods

### Animals, sampling, and phenotypic measurement

The animal care and experimental procedures were approved by the Animal Care Committee of Zhejiang University (Hangzhou, China). Seventeen pairs of mother cows and their respective offspring were selected from a cohort of 361 healthy mid-lactation Holstein dairy cows housed at a commercial dairy farm [6], based on their pedigree records. The animals were reared under the same dairy farm environment and management with a concentrate-to-forage ratio of 57:43 (dry matter basis) [6]. Rumen contents were collected using an oral stomach tube before morning feeding and were used to measure volatile fatty acids (VFAs). Blood samples were collected from jugular venous blood and were stored for genotyping analysis.

### Genotyping detecting the animals’ SNPs

The DNA extraction and genotyping analysis have been described previously [6]. We performed genotyping data analysis in Genome Studio V2.0 software (https://support.illumina.com/array/array_software/genomestudio/downloads.html) and PLINK V1.07 [10]. The genotyping was accomplished through the application of DNA SNP microarray technology, whereby the genotypes were ascertained based on the obtained detection results, facilitating the classification of SNP microarrays according to their genotypic profiles. The genotypes of the SNP were represented by the alleles A, T, C, and G.

### 16S rRNA gene sequencing, rumen microbiota composition, and function analysis

The rumen fluid was collected from two populations of dairy cows (mother population sampled in 2016 and the offspring population sampled in 2019). The 32 species-level rumen heritable bacteria and 674 species-level nonheritable bacteria were obtained from the previously published study and public database (Accession Number PRJNA597489 and PRJNA741384) [6]. For both cohorts from the mother cows (*n* = 254) and the offspring cows (*n* = 107), DNA extraction and 16S rRNA gene amplicon sequencing followed the methodology described previously [2]. Briefly, the DNA of the mother group extracted from each rumen fluid sample was amplified using 341F/806R primer set [11] (5′-CCTAYGGGRBGCASCAG-3′/5′-GGACTACNNGGGTATCTAAT-3′), and genomic DNA of the offspring group was amplified using the 515F/806R primer set (5′GTGY-CAGCMGCCGCGGTAA-3′/5′-GGACTACNVGGGTWTCTAAT-3′). To further identify the long-term effect of host genetics on heritable bacteria, we analyzed the heritable and nonheritable bacteria relative abundance change in 17 pairs of mother cows and their offspring. The presence of batch effect was analyzed and when there was no batch effect, the data were merged into a unified dataset for further downstream analysis. (Figs S1 and S2).

The quality control of the dataset and sequence demultiplexing was performed using QIIME2 v2.0.6 [12] (https://view.qiime2.org) with q2-demux plug-in to demultiplex the original sequence data and DATA2 for denoising. Potential batch effects were ameliorated in the DADA2 pipeline to resolve singleton amplicon sequence variants [13]. SILVA138 database (June 2020 release) was used to map the amplicon sequence variants. PICRUSt2 v2.1.0–b [14] (https://github.com/picrust/picrust2/wiki) software was used to predict microbial functions. The present study quantified microbial functional enrichment using relative abundance percentages as a metric to assess the degree of enrichment.

### Assessment of rumen bacteria stability across generations

Taxa-function stability was used to assess functional stability and defined as the functional shift given a perturbation to the microbiota composition [15]. The relationship between taxonomic perturbation magnitude and functional profile shift was defined as follows:


(1) \begin{equation*} f=\frac{1}{e^a}{t}^b \end{equation*}

where $t$ is the magnitude of the taxonomic perturbation and $f$ is the functional shifts. Two coefficients in the functional shifts, *a* (attenuation) and $b$ (buffering), were used to describe taxa-function stability. Attenuation (*a*) describes the expected rate at which increases in the taxonomic perturbation magnitude are expected to increase functional shifts [15]. Buffering (*b*) indicates how large a perturbation must be before a functional profile shift becomes noticeable and approaches the expected shift magnitude defined by attenuation [15]. Therefore, the higher the *a* and *b* values, the more stable the microbial function. Besides, we used equation (1) to plot the functional response curve and the area under the curve to represent the functional shifts when the taxonomic perturbation happened.

### Network analysis

Correlation networks that indicate the relationship among the 32 rumen heritable bacteria were performed in R software with Spearman’s method. The list of 32 rumen heritable bacteria was compiled based on the previous studies as follows [6]: *p-251-o5*, *Family_XIII_AD3011_group*, *Pirellula*, *Izemoplasmatales*, *Rikenellaceae*, *Ruminococcaceae*, *Monoglobus*, *Defluviitaleaceae_UCG-011*, *[Eubacterium]_hallii_group*, *Anaerovoracaceae*, *Treponema*, *Christensenellaceae*, *Izemoplasmatales*, *Lachnospiraceae, Family_XIII_AD3011_group, Selenomonas_bovis, Atopobium, Pseudoscardovia, wallaby_gut, UCG-002, Lachnospiraceae_NK4B4_group, Lachnospiraceae_XPB1014_group, Clostridia_UCG-014, Roseburia, uncultured_prokaryote, uncultured_Lachnospiraceae, Bacteroidetes_BD2–2, [Eubacterium]_hallii_group, Eubacterium_sp., Flexilinea, Roseburia,* and *Enterorhabdus_sp*. The correlation coefficients with *R* > 0.4 or *R* < −0.4 and *P* < .05 were considered significant. The correlation network was generated by Spearman’s rank correlation and visualized by Cytoscape v3.9.1.

### Statistical analysis

The microbial taxa similarity was calculated based on Bray–Curtis distance method in the R package Vegan [16], and the similarity calculations were based on matrices derived from microbial abundance at both the mother and offspring, as well as the microbial functional abundance profiles. The taxa and function batch effect was removed by R package sva [17]. The analyses of the difference between heritable and nonheritable microbiota in taxa similarity, functional similarity, and functional stability were assessed using the t-test. The analyses of two-generation (mother and their offspring) difference in taxa relative abundance and functional stability were assessed using the paired t-test. The taxa’s relative abundance and rumen’s VFAs among different genotypes of dairy cows were assessed using the nonparametric Kruskal–Wallis test, and we defined the frequency of alleles as the number of different genotypes [18]. All differential analyses were performed using GraphPad Prism V 9.4.1; the significant difference was declared at *P* < .05.

## Results

### Relationship between host genotypes and the relative abundances of heritable bacteria or VFAs concentrations

**Figure 1:**
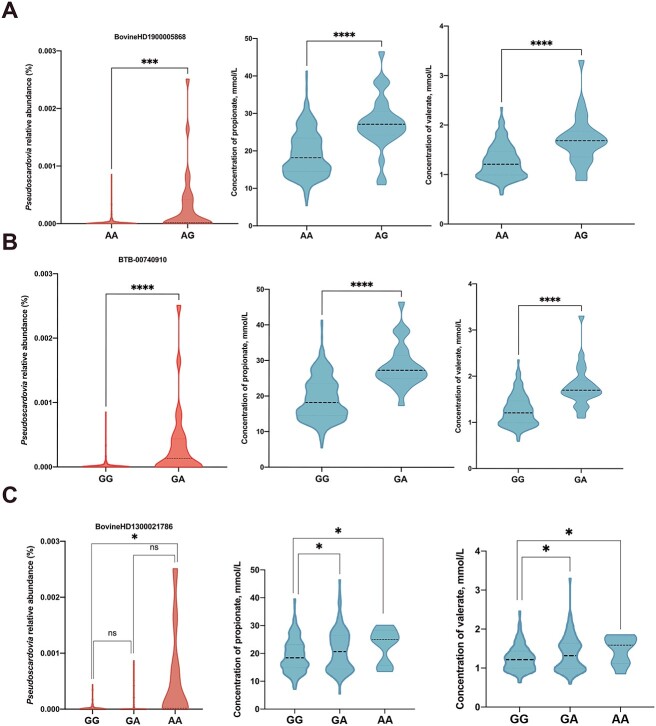
The regulation of host genetics for the relative abundance of *Pseudoscardovia* and rumen’s VFAs. (A-C) The effect of the AG (AA or AG), GA (GG or GA), and GA (GG, GA or AA) genotypes on the abundance of *Pseudoscardovia* and the concentration of propionate and valerate.

**Figure 2:**
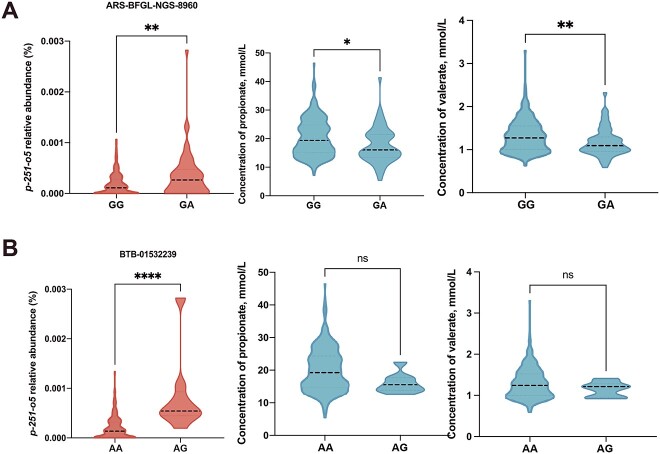
The regulation of host genetics for the relative abundance of *p-251-o5* and rumen’s VFAs. (A-B) The effect of the AG (AA or AG), GA (GG or GA) genotypes on the abundance of *p-251-o5* and the concentration of propionate and valerate.

**Figure 3:**
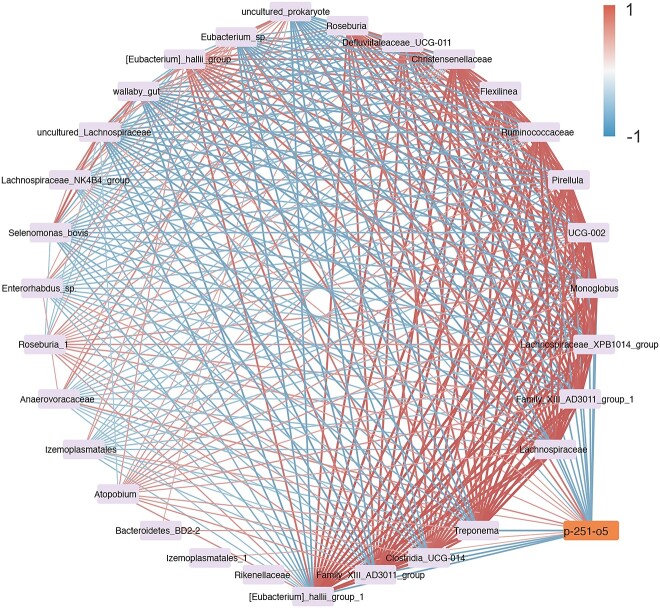
Association analysis between heritable bacteria. A connection represented by a line signifies a positive/negative correlation.

Through genome-wide association analysis to elucidate the relationship between host genotypes and heritable bacteria, or VFAs, we found that five potential single-nucleotide polymorphisms (SNP associated with the relative abundance of 2 heritable bacteria (*Pseudoscardovia*, *p-251-o5*) and concentration of propionate and valerate (Table 1). The frequency of G allele (SNP: BovineHD1900005868) was positively associated with the relative abundance of *Pseudoscardovia* and concentration of propionate and valerate (Fig. 1A). As for SNP: BTB-00740910, the genotype of GA had higher relative abundance of *Pseudoscardovia* and propionate and valerate concentration than the GG genotype (Fig. 1B). The A allele (SNP: BovineHD1300021786) had a significant effect on *Pseudoscardovia* abundance and on propionate and valerate concentrations (Fig. 1C). Despite this, we found that the A allele (SNP: ARS-BFGL-NGS-8960) was positively associated with the *p-251-o5* relative abundance but was negatively associated with the propionate and valerate concentrations (Fig. 2A). The dairy cows with a higher frequency of G allele (SNP: BTB-01532239) exhibited a higher relative abundance of *p-251-o5* and lower concentrations of propionate and valerate (Fig. 2B). *p-251-o5* showed significantly (*P* < .05) high correlations with other heritable bacteria, including *Lachnospiraceae, Family_XIII_AD3011_group_1*, *Lachnospiraceae_XPB1014_group*, *Monoglobus, Eubacterium_sp.*, *UCG-002, Pirellula*, *Izemoplasmatales*, *Ruminococcaceae*, *Flexilinea*, *Lachnospiraceae_NK4B4_group*, *Enterorhabdus_sp.*, *Defluviitaleaceae_UCG-011*, *Roseburia*, *Bacteroidetes_BD2–2*, *Christensenellaceae, Izemoplasmatales_1*, *[Eubacterium]_hallii_group*, *Anaerovoracaceae*, *[Eubacterium]_hallii_group_1*, *Family_XIII_AD3011_group*, *Clostridia_UCG-014*, *wallaby_gut*, *Treponema*, *uncultured_Lachnospiraceae*, *uncultured_prokaryote*, *and Rikenellaceae*. *Pseudoscardovia* didn’t have significant correlations with other heritable bacteria (Fig. 3).

**Table 1:** Identified bovine SNPs influenced heritable rumen microbial taxa abundance.

SNPs	Position	Alleles	Gene	Consequence	Associated taxon	*P* value	*FDR* ^a^
BTB-01532239	14:22781305	A/G	XKR4	intron_variant	*p-251-o5(genus)*	8.29E-10	7.22E-05
ARS-BFGL-NGS-8960	23:14416017	G/A	LRFN2	intron_variant	*p-251-o5(genus)*	3.56E-08	1.03E-03
BTB-00740910	19:17720890	G/A	PSMD11	intron_variant	*Pseudoscardovia(genus)*	6.26E-12	2.73E-07
BovineHD1900005868	19:19945005	A/G	SLC13A2	intron_variant	*Pseudoscardovia(genus)*	1.20E-09	2.62E-05
BovineHD1300021786	13:74704084	G/A	ZNF335	intron_variant	*Pseudoscardovia(genus)*	7.05E-07	3.83E-03

a For each microbial taxonomic feature, *P* value was adjusted into genome-wide false discovery rates (*FDRs*) using the Benjamini-Hochberg method. Associations with *P_adj_* < .01 were considered as significant, and associations with .01 < *P_adj_* < .05 were regarded as suggestively significant.

### The similarity between the mother and offspring in the composition of heritable rumen bacteria

**Figure 4:**
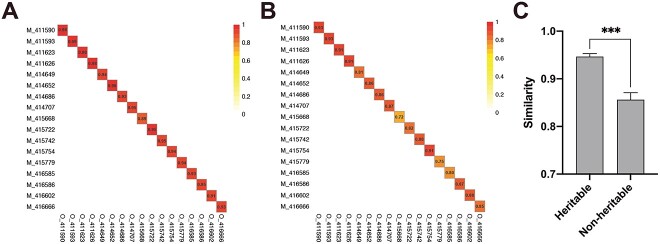
Similarity of heritable and nonheritable bacterial taxa between mothers and their offspring at the species level. (A) Heritable bacteria; (B) nonheritable bacteria. (C) Comparison of the similarity in rumen bacterial composition between heritable and nonheritable bacteria in mother–offspring pairs. Results are presented as mean number of microbial interactions with SE. Indicated *P* values, ^*^P < .05, ^*^^*^P < .005, ^*^^*^^*^P < .0005.

**Figure 5:**
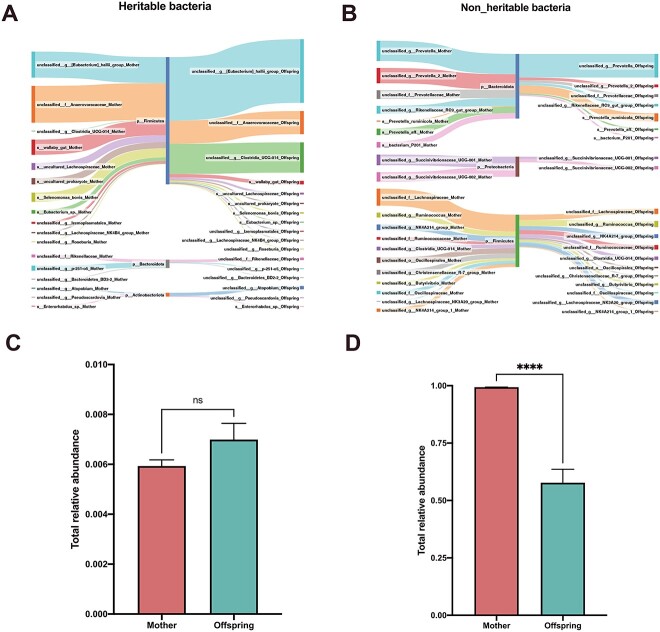
The similarity between the mother and offspring in the composition of heritable rumen bacteria at species level. Sankey diagram depicting the top 20 rumen heritable (A) and nonheritable (B) bacteria flowing through mother to offspring. (C) The difference analysis of total heritable bacteria relative abundance between mother and offspring. (D) The difference analysis of total nonheritable bacteria relative abundance between mother and offspring. Results are presented as mean number of microbial interactions with SE. Indicated *P* values, ^*^^*^^*^^*^P < .0001.

The similarity between mother and offspring was assessed based on calculated values for heritable (Fig. 4A, ranging from 0.89 to 0.99) and nonheritable bacteria (Fig. 4B, ranging from 0.72 to 0.93), with higher mean similarity for heritable bacteria [0.94 ± 0.01; mean ± SE (standard error of mean)] than for nonheritable bacteria (0.85 ± 0.01; mean ± SE) (Fig. 4C). Sankey diagram depicted the top abundant 20 rumen bacteria identified as heritable (Fig. 5A) and nonheritable (Fig 5B) in both mother and offspring. According to our findings, the relative abundance of Firmicutes, which was the most abundant phylum among heritable bacteria, was higher in the offspring’s rumen than in that of their mothers. However, the relative abundance of the phylum Firmicutes in nonheritable bacteria was lower in the offspring compared to their mothers’ rumen nonheritable. In the case of the key heritable bacteria, we identified earlier, which had a significant association between their genotype and the relative abundance of heritable bacteria, the relative abundance of *p-251-o5* in mothers was higher compared to that of their offspring (Fig. 5A and B). In both generations, the heritable bacteria displayed similar relative abundance, with mother cows and their offspring having similar means (0.006 ± 0.0002 [mean ± SE] for mother cows and 0.007 ± 0.0006 [mean ± SE] for daughters), respectively (Fig. 5C), and the relative abundance of nonheritable bacteria reduced from mother (0.994 ± 0.0002; mean ± SE) to offspring (0.577 ± 0.0589; mean ± SE) (Fig. 5D).

### The influence of host genotype on key heritable bacteria in two generations

**Figure 6:**
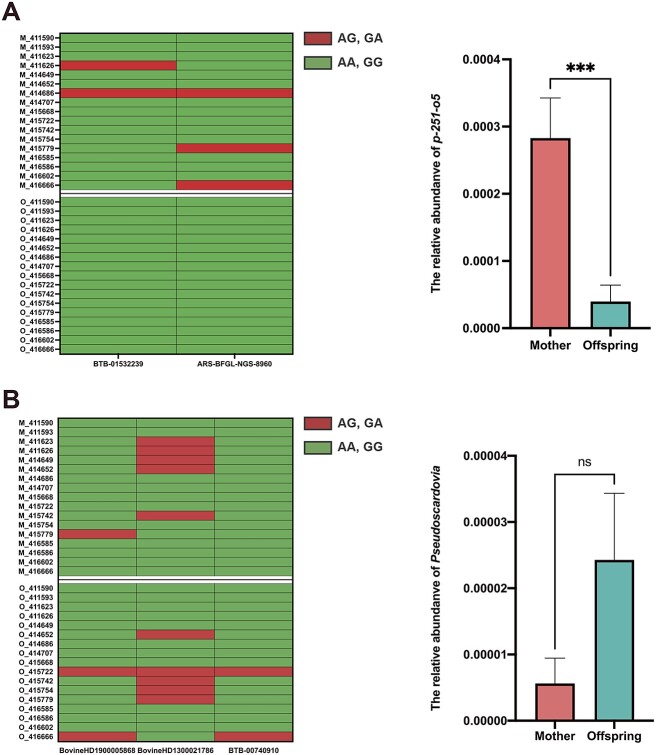
The effect of host genotypes on key heritable bacteria in two generations. (A) The relationship between dairy cows’ genotype and the relative abundance of *p-251-o5*. (B) The relationship between dairy cows’ genotype and the relative abundance of *Pseudoscardovia.* Results are presented as the mean number of microbial interactions with SE. Indicated *P* values, ^*^^*^^*^P < .0005.

Across generations, the A allele (SNP: ARS-BFGL-NGS-8960) and G allele (SNP: BTB-01532239) were significantly associated with the relative abundance of *p-251-o5* (Fig. 6A). The mother group had more A allele and G allele than the offspring group, and the relative abundance of *p-251-o5* showed a significant difference between mother and offspring (Fig. 6A). The SNP (SNP: BovineHD1900005868, SNP: BTB-00740910, SNP: BovineHD1300021786) genotypes were not different between mother and offspring, and the relative abundance of *Pseudoscardovia* was not different between mother and offspring (Fig. 6B).

### The effects of the mother’s genotypes on the functions of heritable rumen bacteria and their stability in offspring

**Figure 7:**
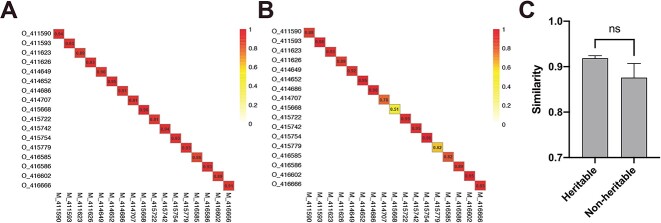
The effects of the mother’s genotypes on the function of heritable rumen bacteria in offspring. The functional similarity between mother and offspring is the heatmap of heritable (A) and nonheritable (B). Zero represents the rumen bacterial function totally different between mother and offspring; 1 represents the mother and offspring having the same rumen bacterial function. (C) The mother and offspring rumen bacterial function similarity differences between heritable and nonheritable bacteria. Results are presented as the mean number of microbial interactions with SE.

**Figure 8:**
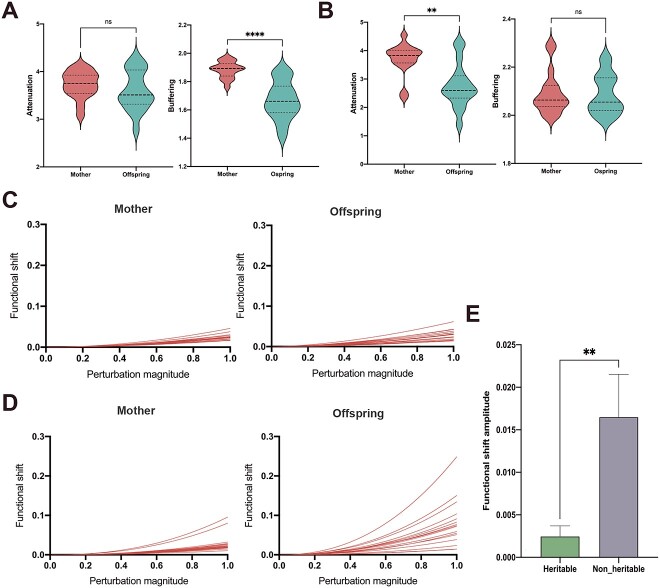
Transmission of heritable and nonheritable bacterial functional stability from mother to their offspring. (A) Comparison of heritable bacterial function attenuation and buffering between mother and their offspring. (B) Comparison of nonheritable bacterial function attenuation and buffering between mother and their offspring. Left figures C and D represent the mother cows’ functional shift, and right figures C and D represent the offspring cows’ functional shift. (E) The function stable ability difference between heritable and nonheritable bacteria. Results are presented as the mean number of microbial interactions with SE. Indicated *P* values, ^*^P < .05, ^*^^*^P < .005, ^*^^*^^*^P < .0005, ^*^^*^^*^^*^P < .0001.

In total, 191 and 236 predicted biological functions were obtained for heritable and nonheritable bacteria, respectively. The functional similarity and functional stability of heritable and nonheritable bacteria were analyzed between mother cows and their offspring. The functional similarity was calculated between mother and offspring of both heritable (Fig. 7A, ranging from 0.87 to 0.96) and nonheritable (Fig. 7B, ranging from 0.51 to 0.98). There was no significant difference between mother and offspring (Fig. 7C). For heritable bacteria, the offspring had lower buffering value than mother cows, but no difference was found in attenuation value between two generations (Fig. 8A). However, the offspring had lower attenuation value in nonheritable bacteria than mother cows, with no difference found for buffering index (Fig. 8B). We estimated the functional shift amplitude for heritable and nonheritable bacteria (Fig. 8C, D) and found that heritable bacteria had higher functional stability than nonheritable ones (Fig. 8E).

## Discussion

Our previous study has found 32 heritable and 674 nonheritable bacteria in the rumen of Holstein dairy cows [6]. Building on this study, our current research provides additional evidence on how host genotype influences the abundance of rumen bacteria. Our findings uncovered two rumen heritable bacteria (*p-251-o5*, *Pseudoscardovia*), which their abundances are associated with host genotypes, and several genotypes (AA, GG, GA, AG) had different potentials to affect the relative abundance of heritable bacteria. Similarly, Yang et al. [18] mapped a quantitative trait locus that were significantly associated with the abundance of *Erysipelotrichaceae* species in pigs and found the frequency of the A allele (AO gene) increased the *Erysipelotrichaceae* abundance. Their study focused on monogastric animals, while our current study demonstrates that a similar relationship can exist in ruminants, suggesting that the potential to manipulate rumen heritable bacteria via host genotype modulation. The SNP: BTB-01532239 located in leucine-rich repeat and fibronectin type III domain containing 2 (*LRFN2*) gene associated with the relative abundance of the bacterial species belonging to the unclassified genus of *p-251-o5*. The function of this gene has been found to be involved in the modulation of chemical synaptic transmission and regulation of postsynapse organization [19], indicating that the host has the potential to modulate the abundance of rumen bacteria that exhibit heritability through the detection of signaling molecules present in the rumen. However, research is needed to determine what signal molecules would influence relationship between *LRFN2* and *p-251-o5*. The SNP: BovineHD1900005868 (had significant effects on the abundance of the unclassified genus of *Pseudoscardovia*) is located within the gene *SLC13A2* (solute carrier family 13 member 2) on BTA 19. The plasma membrane transporter *SLC13A2*, a member of the Na + −dependent SLC13 family, exhibits high sensitivity toward succinic acid and is capable of transporting it [20] and has been reported to be involved in intestinal host–microbe interactions in mice [21]. Slawinska et al. [22] found that the *SLC13A2* takes part in pathways involved in the transport of glucose and other sugars amine compounds. Although the function of the gene *SLC13A2* has not been well studied in cattle, it is important to study the genetic variation of this gene and its expression of this gene in the rumen epithelial tissue. This suggests that the host genetics–based microbial regulation strategy (sensing and transporting small molecules) can be a potential mechanism for efficiently regulating host–microbiome interaction in the rumen.

We further delineated bacterial composition similarity in mother cows and their offspring. Here, we found that heritable bacteria had greater bacterial composition similarity than the nonheritable bacteria between mother cows and their offspring. Lima et al. [9] showed that bovine calf fecal microbiota clustered closely to the maternal fecal microbiota, and Zhu et al. [24] provided evidence that the fecal microbiome of the calf may be originated from maternal sources. According to Guo et al. [25], the rumen microbiota of calves showed significant similarity to that of cows during the first 2 weeks of life. Specifically, the majority of the microbiota found in the rumen of calves was derived from that of mother cows. The findings indicate that the maternal influence on the microbial community of the offspring is substantial. When comparing the host genetic effects on heritable bacteria in the rumen between two generations, it was observed that there is a significant impact of host genetics on shaping the *p-251-o5*, *Pseudoscardovia* relative abundance. Moreover, our findings suggest the effects of the mother’s genotypes on the composition of heritable rumen bacteria in offspring. Jewell et al. [26] revealed that the ruminal bacterial community is dynamic in terms of composition and diversity during two lactation cycles. The abundance of heritable bacteria exhibited variability across two generations, implying the potential for modulating heritable bacteria through genetic breeding approaches. In addition, according to a previous study [6], the *p-251-o5* has high (h^2^ = 0.79 ± 0.09; mean ± SE) heritability estimate, which offers a prospective breeding strategy for rumen bacteria of dairy cows in future.

It is also known that the changes in rumen bacteria composition may not lead to the function and functional stability change. The analysis of the functional similarity and stability of heritable and nonheritable bacteria revealed no difference in functional similarity between heritable and nonheritable bacteria, and the functional similarity had a higher value than the composition similarity between the two generations. Zhu et al. [27] sampled the rumen fluid within the same lactation with a 122 days interval and found that the rumen bacterial communities and functions of dairy cows displayed distinct stability at different taxonomic levels. These findings suggest that rumen bacterial functions maintain a relatively stable state over time, indicating higher stability in functions compared to compositional changes. Furthermore, we observed that heritable bacteria exhibited greater functional stability compared to nonheritable bacteria, as supported by previous research indicating that heritable microbes act as central nodes within microbial interaction networks [5]. Li et al. [4] demonstrated the keystone status of heritable microbial taxa in the rumen microbial correlation network, while other study [5] emphasized the pivotal role of heritable bacteria in rumen bacterial interactions. Based on these findings, we postulate that the high stability of heritable bacteria may be attributed to their influential position in the microbial network. The current study also has limitations in terms of its research on functional prediction, and future research can explore the function of rumen microbiota based on metagenomics. Our study has provided a novel direction for rumen bacterial research, focusing on genetically selected heritable bacteria and better lactation performance for cows.

In our study, we analyzed the relationship between host genotypes and heritable bacteria or VFAs and found that five potential SNP genotypes were associated with the relative abundance of two heritable bacterial (*Pseudoscardovia*, *p-251-o5*) and concentrations of two VFAs (propionate, valerate). Furthermore, our previous study [6] also revealed significant associations between *p-251-o5* and the concentrations of propionate and valerate in the rumen, as well as significant associations between *Pseudoscardovia* and acetate, propionate, and valerate concentrations within the rumen. The mother–offspring comparison revealed that the heritable rumen bacteria had higher similarity than nonheritable bacteria between two generations (mother cows and their offspring), and the predicted functional analysis showed that heritable microbial functions had higher stability than the nonheritable bacteria. It is noticeable that due to the considerable time span covered by this study, there were variations in the primers employed between the two experimental periods. However, Claesson et al. [23] indicate that these two sets of primers exhibit similar accuracy in bacterial identification, with limited disparities. Furthermore, our study was subjected to batch effect analysis for both sets of data to maximize data consistency. Despite with such limitations, our results have revealed the evidence that the host genotypes can have a potential effect on rumen microbial relative abundance. Understanding the stability of the heritable and nonheritable bacterial composition and function will provide a scientific view to developing precision manipulation strategies of rumen heritable microbial groups to improve cattle production traits. Our findings highlight an opportunity to manipulate heritable bacteria to improve cow’s performance.

## Supplementary Material

Figure_S1_ycad020

Figure_S2_ycad020

## Data Availability

The rumen bacteria raw data for our samples are from the NCBI under accession numbers: PRJNA597489 and PRJNA741384. The SNP data have been deposited in the Genome Variation Map (G.V.M.) in China National Center for Bioinformation, under accession numbers PRJCA004923.
