# Supplementary material for: Genotype-associated heritable rumen bacteria can be a stable microbiota passed to the offspring
Source: ISME Commun. 2024 Jan 10;4(1):ycad020. doi: 10.1093/ismeco/ycad020 (PMC10848306; doi:10.1093/ismeco/ycad020)
Supplement: Figure_S1_ycad020 [file figure_s1_ycad020.pdf]

FIG S1

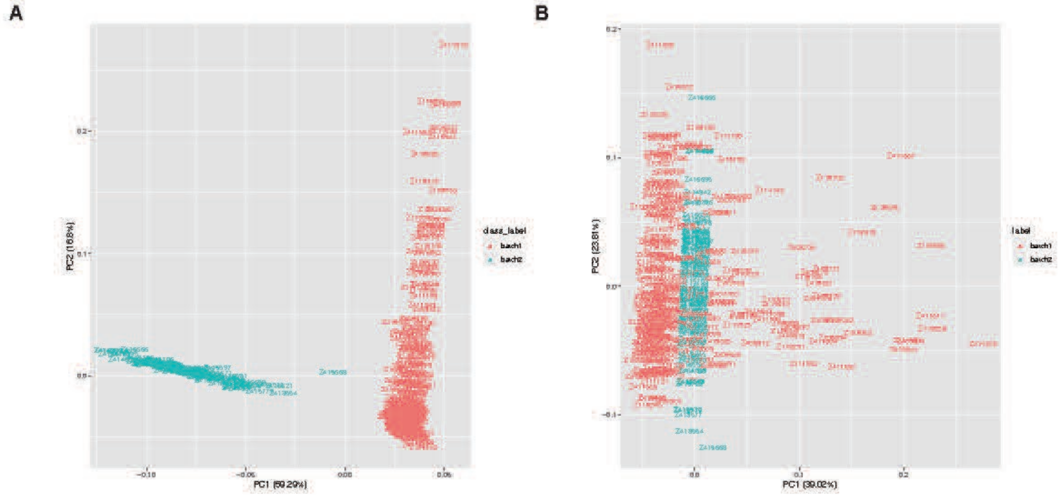

FIG S1. Principal component analysis of rumen bacteria before (A) and after (B) batch effect adjustment based on the remove batch effect method.
