# Supplementary material for: Genotype-associated heritable rumen bacteria can be a stable microbiota passed to the offspring
Source: ISME Commun. 2024 Jan 10;4(1):ycad020. doi: 10.1093/ismeco/ycad020 (PMC10848306; doi:10.1093/ismeco/ycad020)
Supplement: Figure_S2_ycad020 [file figure_s2_ycad020.pdf]

FIG S2

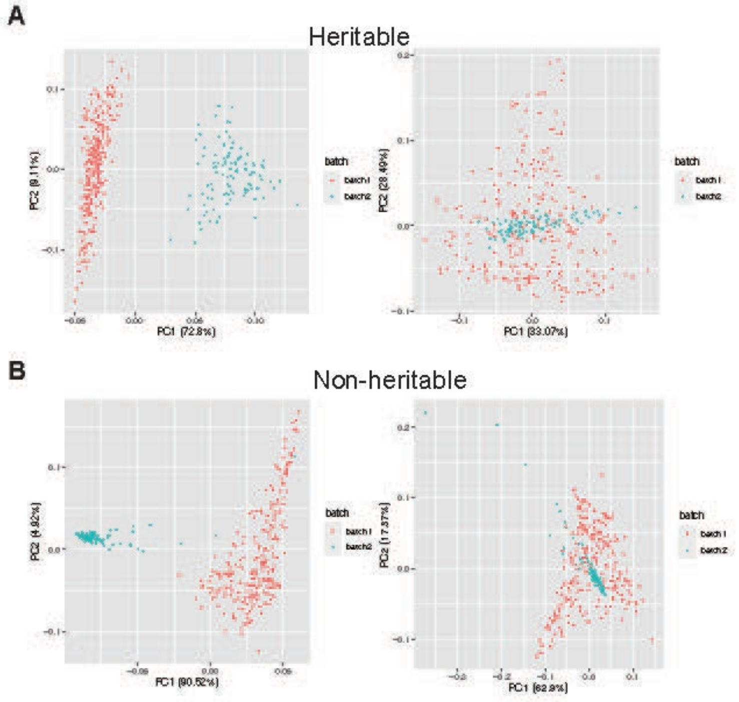

FIG S2. Principal component analysis of rumen heritable (A) and non-heritable (B) bacteria function before (left figure A, B) and after (right figure A, B) batch effect.
